# A whole rock geochemical dataset for magmatic rocks drilled on the mid-Norwegian margin

**DOI:** 10.1038/s41597-026-07073-x

**Published:** 2026-03-24

**Authors:** C. Tegner, P. Guo, S. Chatterjee, S. Lambart, M. T. Jones, S. Planke, H. H. Svensen, E. R. Neumann, C. E. Lesher, K. Cashman, T. Takahashi, E. H. Cunningham, A. M. Morris, E. W. Stokke, J. M. Millett, G. T. F. Mohn, J. Longman, C. Berndt, C. A. Alvarez Zarikian, P. Betlem, E. C. Ferré, I. Y. Filina, J. Frieling, D. T. Harper, R. P. Scherer, N. Varela, W. Xu

**Affiliations:** 1 Department of Geoscience, Aarhus University, Aarhus, Denmark; 2 Institute of Oceanology, Chinese Academy of Sciences, Qingdao, China; 3 Earthquake Research Institute, The University of Tokyo, Bunkyō, Tokyo, Japan; 4 Department of Geology and Geophysics, University of Utah, Salt Lake City, UT USA; 5 Department of Ecology, Environment and Geoscience, Umeå University, Umeå, Sweden; 6 Department of Geosciences, University of Oslo, Oslo, Norway; 7 Volcanic Basin Energy Research AS, Høienhald, Oslo, Norway; 8 Department of Earth and Planetary Sciences, University of California, Davis, CA 95616 USA; 9 Department of Earth Sciences, University of Oregon, Eugene, OR 97403-1272 USA; 10 Niigata University, Niigata, Japan; 11 Department of Geology and Geophysics, University of Aberdeen, King’s College, Aberdeen, UK; 12 Laboratoire Géosciences et Environnement Cergy, CY Cergy Paris Université, Cergy, France; 13 School of Geography and Natural Sciences, Northumbria University, Newcastle Upon Tyne, UK; 14 GEOMAR Helmholtz Centre for Ocean Research Kiel, Kiel, Germany; 15 Scientific Ocean Drilling Coordination Office, Texas A&M University, College Station, USA; 16 Norwegian Geotechnical Institute, Oslo, Norway; 17 The University Centre in Svalbard, Longyearbyen, Norway; 18 Department of Geological Sciences, New Mexico State University, Las Cruces, NM USA; 19 Department of Earth and Atmospheric Sciences, University of Nebraska, Lincoln, NE USA; 20 Department of Earth Sciences, University of Oxford, Oxford, UK; 21 Department of Earth, Atmosphere and Environment, Northern Illinois University, DeKalb, IL USA; 22 Department of Environmental Sciences, University of Virginia, Charlottesville, VA USA; 23 School of Earth Sciences and the Research Ireland Centre for Applied Geosciences, University College Dublin, Dublin, Ireland

## Abstract

The mid-Norwegian margin is one of the best studied volcanic rifted margins on Earth. Geophysical investigations have demonstrated the presence of well-developed inner and outer Seaward Dipping Reflectors (SDRs), landward flows, lava deltas, marginal highs, volcanic centers, ash layers, and sill complexes. These features have been proven to consist of magmatic rocks through the international Deep Sea Drilling Program (DSDP Leg 38, 1974), Ocean Drilling Program (ODP Leg 104, 1985), International Ocean Discovery Program (IODP Expedition 396, 2021), and commercial drilling. A total of fifteen drill cores penetrated magmatic rocks that formed between 57 and 50 million years ago (Ma). Here we provide (i) new (*n = *224) major and trace element compositions obtained by X-ray fluorescence (XRF), inductively-coupled plasma mass spectrometry (ICP-MS), and inductively-coupled optical emission spectrometry (ICP–OES) on whole rock powders of magmatic rocks for IODP Exp. 396 (*n* = 119), ODP Exp. 104 (*n = *79), DSDP Exp. 38 (*n* = 24); and (ii) a compilation of all new and published data for magmatic rocks in the fifteen drill cores (*n* = 563). Portable X-ray fluorescence (pXRF) data (*n* = 381) for the IODP Exp. 396 cores are also reported. These datasets provide a resource for examining the origin of magmatism associated with continental breakup and rifted margin formation, particularly the formation of excess magmatism compared to normal mid-oceanic spreading ridges, mantle-crust interaction, and the linkage of magmatism to global hyperthermal events on Earth’s surface.

## Background & Summary

**Fig. 1:**
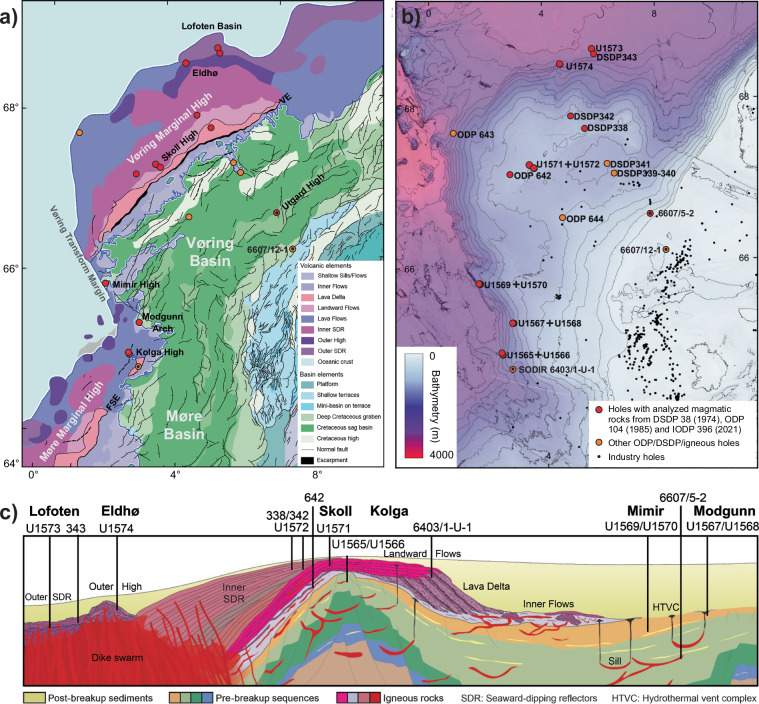
Structural elements (**a**) and bathymetric map (**b**) showing the drill cores with magmatic rocks on the mid-Norwegian margin. A total of 15 sites was drilled during DSDP Expedition 38 (1974), ODP Expedition 104 (1985), IODP Expedition 396 (2021), and commercial drilling. See also Table 1. (**c**) Generally accepted cross section of the region showing the volcanic structures of the Vøring Basin and Marginal High, and approximate projections of the rocks penetrated by the drill cores. Modified from reference^8^.

The opening of the Northeast Atlantic initiated around 56 Ma and was associated with massive magmatic activity forming the North Atlantic Igneous Province (NAIP)^1,2^. On the mid-Norwegian margin these magmatic rocks cover two topographic highs on the continental shelf named the Vøring and Møre Marginal Highs (Fig. 1a,b)^3^. The NAIP forms one of the largest large igneous provinces (LIPs) on Earth, offering unique insights into the magmatic processes that accompany continental break-up^4,5^. The mid-Norwegian Margin has therefore been targeted on three occasions by three generations of the scientific ocean drilling programs^6–17^. In addition, commercial drilling for hydrocarbons has encountered intrusive complexes associated with the province in the neighboring basins^18^. A total of 15 drill sites has penetrated the magmatic rocks on the mid-Norwegian Margin as summarised in Table 1, with their locations shown in map view (Fig. 1a,b) and in a schematic cross-section (Fig. 1c). This publication provides a compilation of new and published major and trace element compositions measured on 563 whole rock samples of magmatic rocks from the 15 drill cores. Also included are 381 calibrated portable X-ray fluorescence (pXRF) analyses on samples from the IODP Exp. 396 drill cores.

**Table 1:** Summary of boreholes with geochemical analyses of Paleogene igneous rocks on the mid-Norwegian Margin included in this contribution.

Site*	Location	Magmatic rocks	Data types	References^§^
*IODP 396 (2021)*				
U1565	Kolga High, Møre Basin	Granite (c. 7 m)	WR	10, 19, 27
U1566	Kolga High, Møre Basin	Granite (c. 27 m) overlain by basaltic flows and sediments (c. 129 m)	WR, pXRF	11, 19, 27
U1567	Modgunn Hydrothermal Vent Complex, Vøring Basin	Basaltic ash layers in Eocene mudstones filling the vent structure	WR	12, 17, 19, 27
U1568	Modgunn Hydrothermal Vent Complex, Vøring Basin	Basaltic ash layers in Eocene mudstones filling the vent structure	WR	12, 17, 19, 27
U1569	Mimir High, Vøring Basin	Basaltic ash layers in Eocene sediments	WR	13, 19, 27
U1570	Mimir High, Vøring Basin	Basaltic ash layers and 2 pyroclastic dacites in Eocene sediments	WR	13, 19, 27, 35
U1571	Vøring Margin (Inner SDRs)	Basaltic flows (c. 100 m); minor occurences of ash and hyaloclastite	WR, pXRF	14, 19, 27
U1572	Vøring Margin (Inner SDRs)	Basaltic flows (c. 120 m); minor occurences of ash and hyaloclastite	WR, pXRF	14, 19, 27
U1573	Vøring Margin, Lofoten Basin (Outer SDRs)	Basaltic flows (c. 48 m)	WR, pXRF	15, 19, 27
U1574	Vøring Margin, Eldhø (Outer High)	Basaltic flows and hyaloclastite (c. 94 m)	WR, pXRF	16, 19, 27
*Esso (1991; stored at Norwegian Petroleum Directorate)*				
6607/5-2	Vøring Basin, Utgard	Two doleritic sills (91 m and >50 m, respectively)	WR	18, 27, 34
*ODP 104 (1985)*				
642E	Vøring Margin (Inner SDRs)	Basaltic flows, dykes and sills; dacitic flows and pyroclastics (c. 900 m)	WR	7, 19, 27, 31-33
*DSDP 38 (1974)*				
338	Vøring Margin (Inner SDRs)	Basaltic flows (c. 19 m)	WR	6, 19, 27-30
342	Vøring Margin (Inner SDRs)	Basaltic flows (c. 12 m)	WR	6, 19, 27-30
343	Vøring Margin, Lofoten Basin (Outer SDRs)	Basaltic flows (c. 30 m)	WR	6, 19, 27-30

Abbreviations: WR = Whole rock data based on analyses of powder; pXRF = portable XRF analyses diectly on the surface of drill core.

*GPS positions are listed in the data files (references^19,27^).

^§^Ref. ^19^ is new data reported here and reference^27^ is data included in the data compilation file.

## Methods

Here we describe the methods for new data on whole rock powders (n = 224) and new portable XRF (pXRF) measurements (*n* = 381) on drill core surfaces, and published here for the first time^19^.

The whole rock powder data involved sample preparation and analyses between six laboratories as described below and in ref. ^19^. The samples were taken from the working halves of drill cores aiming for the freshest rock possible, avoiding hydrothermal veins, and representing the variety of the igneous units.

### Crushing and powdering

At Aarhus University (Denmark) a subset of drill core samples (n = 72) was crushed to small aggregates (<2 cm) in a hydraulic steel press and c. 25 g were subsequently powdered in a corundum shatterbox^20^. At the Geological Survey of Denmark and Greenland (GEUS, Denmark) samples (n = 79) were powdered in a tungsten carbide ball mill^21^. The ash samples (n = 13) were prepared and powdered in an agate mortar at the University of Oslo (Norway). The remaining new samples (n = 60) were powdered at the Institute of Oceanology, Chinese Academy of Sciences (IOCAS) in China. After saw grinding off the marks and pen marks, samples were crushed into 1–2 cm fragments using an agate mortar. The fragments were then ultrasonically cleaned in Milli-Q water, dried, and powdered into 200-mesh using an agate mill.

### Bulk rock major element analyses

At Aarhus University the major element compositions were determined by X-ray fluorescence (XRF) on fused glass discs prepared and analyzed as described previously^22^. Concentrations of FeO were determined by titration with potassium dichromate and the mass lost on ignition (LOI) by heating the powder in air in a muffle furnace at 950 °C for 3 h. At the Institute of Geochemistry, Chinese Academy of Sciences (IGCAS), bulk rock major element analysis was done using a Thermo Scientific ARL X-ray fluorescence (XRF) spectrometer. At GEUS, the major elements were determined by XRF^21^. Finally, at Niigata University (Japan), the major elements were determined using XRF spectrometry (Rigaku RIX3000) following established analytical methods^23^, with an optimisation for ultramafic rocks^24^. The major element compositions of the ash samples were determined at Actlabs, Ontario, Canada, using lithium metaborate/tetraborate fusion of the rock powder followed by dissolution in 5% HNO_3_ and analysis by inductively coupled plasma – optical emission spectrometry (ICP–OES) and mass spectrometry (ICP–MS).

### Bulk rock trace element analyses

The trace element compositions were determined by inductively coupled plasma mass spectrometry (ICP-MS) in four laboratories. At GEUS, the sample powders were dissolved in a mixture of HNO_3_ and HF and analyzed using a PerkinElmer Eland 6100 DRC quadrupole calibrated against international standards^21^. At IOCAS, bulk rock trace elements were analyzed using an Agilent-7900-MS^25^. In brief, approximately 50 mg of each sample was dissolved with an acid mix of double-distilled concentrated HCl + HNO_3_ (a 3:1 by volume mixture of HNO_3_ and HCl) and HF in a high-pressure bomb for 15 hours. The solution was then re-dissolved in distilled 20% HNO_3_ for 2 hours till complete digestion/dissolution. Time-drift correction and quantitative calibration were conducted using the in-house software ICPMSDataCal. Finally, at Niigata University, the bulk trace element concentrations were determined using a Yokogawa HP4500 ICP-MS, following the acid digestion^26^. For each sample, 100 mg of material was weighed and placed in a Teflon vial and underwent step-by-step acid treatment with heating until the sample reached complete dissolution. The solution was finally diluted using HNO_3_ and an internal standard (^115^In) was measured along with secondary standards (BHVO-2, W2a and JB2). The trace element composition of the ash samples was determined by Actlabs using ICP-MS on a diluted aliquot of the solution used for major element analysis. Trace elements Ba, Be, Sc, Sr, V, Y, and Zr were measured by ICP-OES.

### Portable XRF (pXRF)

Individual *in situ* XRF analyses were acquired on board D/S JOIDES Resolution during IODP Exp. 396 using an Olympus Delta handheld portable XRF spectrometer. Details on analysis acquisition are provided in ref. ^9^ and the pre-calibration data are reported in the IODP LIMS database (https://web.iodp.tamu.edu/LORE/). Measurements were carried out directly on the split core surface. The instrument was operated in the “geochemistry” mode, which employs three sequential beam settings to optimise detection across the elemental spectrum. These settings target elements in three distinct energy ranges (low = Al, Si, K, Ca, Ti, Mn, Fe, Cr, P, S, and Mg; main = Ca, Ti, Mn, Fe, Ni, Sr, Rb, Zr, Zn, and others; high = Sr, Rb, Zr, Mo, Ag, Cd, Sn, W, Hg, Pb, Bi, Th, and U). Thus, three analyses were performed per data point. A powder-mounted standard reference material (BHVO-2) was analyzed every 8–10 measurements to track instrument performance over time. No instrument drift was observed with time.

In addition to the internal calibrations of the instrument, calibration curves for the different elements measured via pXRF were determined using a suite of standard reference materials (JP-1, BE-N, BIR-1. BHVO-2, BCR-2, JB-2, DTS-1, MRG-1, AVG-1, JG-1a, JA-2, JR-1, LKSD-4, and NODA-1) chosen to cover the expected range of compositions for the analyzed samples. The equations for each element and their associated R^2^ values can be found in the “Method Specific Metadata”^19^.

Following the approaches established during IODP Expeditions 352 and 366, we only report average corrected values for elements with calibration curves exhibiting high correlation coefficients (R^2^ > 0.95). Consequently, P, Nb, and V are only considered as qualitative and are not reported in this database. Molybdenum and Hg were also excluded as they were not present in the standards used for calibration. In addition, Si, Mg, and Al produce the lowest energy X-rays that are readily absorbed in air. Hence, even if their calibration curves are good, we do not report the corrected values as these concentrations should be treated as semi-quantitative at best. Finally, analyses of Ag, Cd, Sn, W, Pb, Bi and Th systematically fall below the limit of detection. The remaining elements with reliable calibrations are Ca, K, Ti, Fe, Mn (calibrated as oxides: CaO, K_2_O, TiO_2_, FeO^T^, and MnO), Ni, Cr, Cu, Zn, Sr, Rb, Zr, and Y.

## Data Records

Two separate datasets are available in the GEOROC repository hosted by GFZ Data Services^19,27^. One dataset provides new whole rock data on powder material (*n = *224) and pXRF data measured on the slip core surface (*n* = 381). This dataset can be downloaded as an excel file (file name: *2025-011_Tegner-et-al_mid-Norwegian-Margin-Data_Earthchem_Template.xlsx*) from this link 10.5880/digis.2025.011^19^. This file also provides details of the core pieces analysed and a summary of analyses of international reference standards and laboratory house-standards analyzed together with the unknowns.

The second dataset provides a compilation of 563 entries of bulk rock major and/or trace element data, including published as well as the newly acquired whole rock powder data. This dataset is given as an *Expert Dataset* in the GEOROC repository and can be downloaded as an excel file (file name: Expert dataset: A whole rock geochemical dataset for magmatic rocks drilled on the mid-Norwegian margin) via this link 10.5880/digis.e.2025.005^27^.

## Data Overview

This section provides a brief overview of the whole rock composition dataset, the pXRF data, and outlines their potential use and significance.

### Bulk rock major and trace element compositions measured on rock powders

The GEOROC *Expert Dataset*^27^ represents a compilation of all major and/or trace element analyses (*n* = 563) of igneous rocks drilled on the mid-Norwegian margin. This compilation is divided into published legacy data and newer data from our group. The legacy data (*n* = 317) includes compositions for DSDP Exp. 38 drill Sites 338, 342 and 343^28–30^ and for ODP 104 Site 642E^31–33^. The newer data (*n* = 246) includes (i) published data for two sill intrusions (Utgaard sills) emplaced into the Vøring Basin drilled by Esso (1991) and cuttings are stored at the Norwegian Petroleum Directorate^18,34^ (*n* = 22), (ii) published data for pyroclastic deposits of dacitic composition cored at Site U1570^35^ (*n* = 2), and (iii) new data (n = 224) for IODP Exp. 396 Sites U1565–U1574^10–16^, ODP 104 Hole 642E and DSDP Sites 338, 342 and 343^19^. Some of the data for IODP Exp. 396 are discussed in detail in a companion paper^36^.

**Fig. 2:**
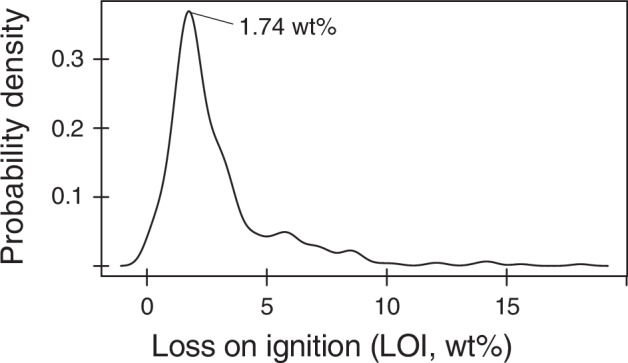
Kernel density distribution of loss-on-ignition (LOI) values (n = 416).

One challenge with igneous rocks from the ocean floor is to discern effects of alteration from primary compositions^37^. A first-order evaluation of alteration is the weight loss-on-ignition (LOI) that has been shown to correlate positively with the mode of hydrous phases formed by hydrothermal alteration and interaction with seawater^38,39^. Basalts with less than 2 weight percent (wt%) LOI are typically considered unaltered^40^, but rocks with up to 4 wt% LOI have also been argued to largely retain their primary, igneous compositions^39,41^. The density distribution of available LOI values (n = 416) for the entire dataset shows a peak at 1.74 wt% LOI (Fig. 2) and 81% of the samples have values below 4 wt%. The highest LOI value is 18 wt%. We therefore conclude that most samples represent largely unaltered igneous compositions, but effects of alteration should be considered carefully for rocks with elevated LOI values.

**Fig. 3:**
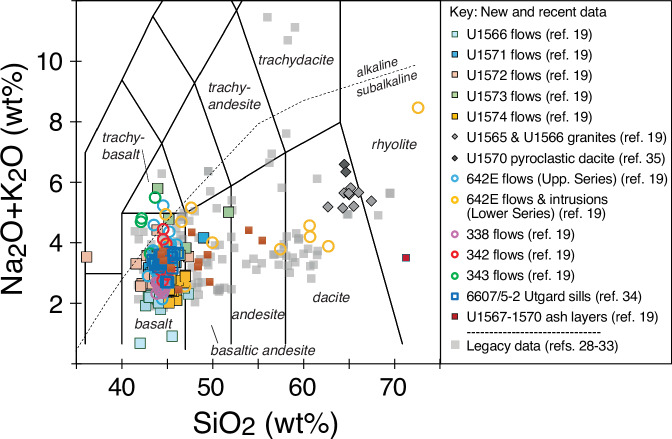
TAS diagram for all data on the mid-Norwegian margin listed in the data compilation^27^. References are given in the reference list. Classification fields from ref. ^42^.

**Fig. 4:**
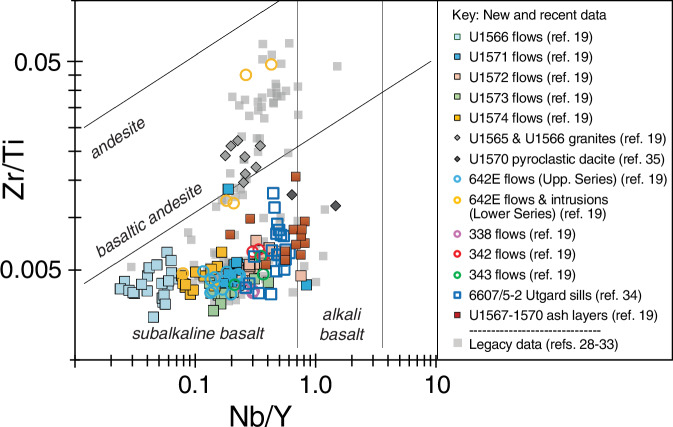
Zr/Ti vs Nb/Y for all data on the mid-Norwegian margin listed in the data compilation^27^. References are given in the reference list. Classification fields from ref. ^43^.

The total alkali vs SiO_2_ (TAS) diagram (Fig. 3) outlines the range of all igneous rock compositions measured from the mid-Norwegian margin. This shows that the dominant composition of lavas, ashes, dykes, and sills is subalkaline basalt with a few data points plotting in the basaltic andesite and trachybasalt fields. A few of the basalts plot above the alkaline-subalkaline subdivision line^42^. However, the Nb/Y values are mostly below 0.8 (Fig. 4) and this ratio of immobile trace elements is considered strong evidence that the rocks are predominantly subalkaline^43^. The dataset also demonstrates the occurrence of silicic rocks at four drill sites (Fig. 3). This includes dacite to rhyolite compositions encountered in flows, volcaniclastic sediments and dykes in the basal part of Hole 642E and denoted the Lower Series^31,32,44^. The recent IODP Exp. 396 drilling further penetrated silicic rocks in the form of granites in the lower portions of Sites U1565^10^ and U1566^10,11^, pyroclastic deposits of dacitic composition with garnet and cordierite minerals at Sites U1569 and U1570^13,35^, and in one ash layer at Site U1567^12^.

### *In situ* major and trace element compositions measured on split core surfaces (portable XRF)

**Fig. 5:**
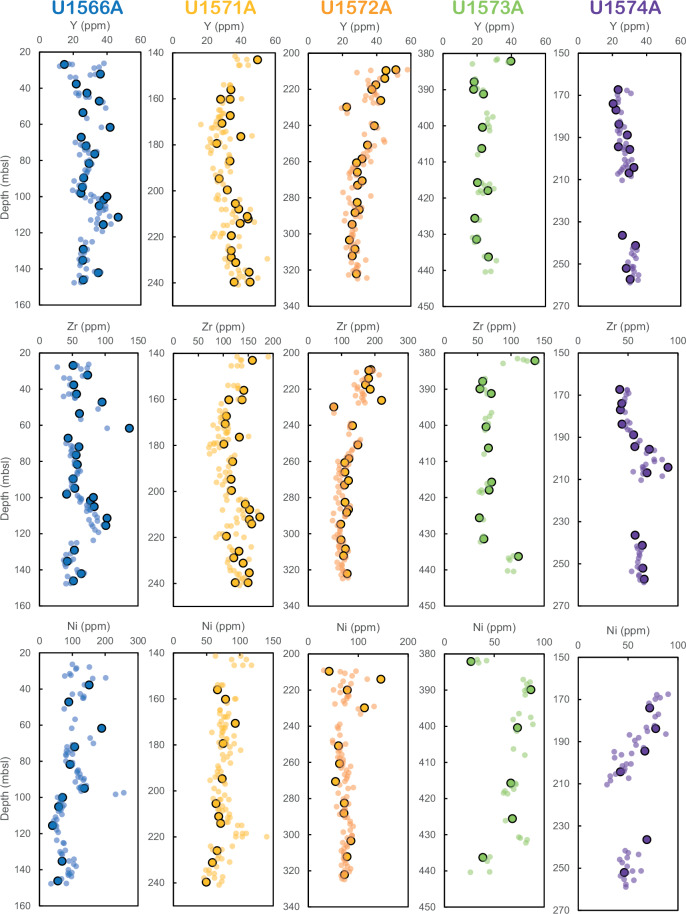
Chemical stratigraphy obtained by pXRF measurements (small symbols) on the basalts collected at the various sites during IODP Exp. 396 and compared with the ICP-MS analyses performed on bulk rocks (large symbols). mbsl = meters below sea level. Data are available in ref. ^19^.

The calibrated data measured by pXRF directly on the split surface of the core material^19^ can become very valuable for characterisation of extrusive rocks when these are dominated by aphyric basalts for which petrographic variations might be minimal, as encountered during IODP Exp. 396. In addition, they also provide a rapid and non-destructive chemical characterisation that allows for producing a high spatial resolution dataset in comparison to the more time consuming and more expansive discrete whole rock analyses. Figure 5 presents a comparison of the chemical stratigraphy between sites from IODP Exp. 396 and demonstrates consistency between pXRF and ICP-MS data. Moreover, it shows that basalts collected at Site U1566 have smaller-scale variations, consistent with core observations demonstrating that the igneous units are composed of multiple thin lava flows^11^, while other sites display more massive and thicker igneous units.

### Use and significance

The datasets can be used to test hypotheses for the origin of excess magmatism during continental rifting and the development of volcanic-rifted margins^4,8,45^. For example, the whole-rock compositions can provide constraints on mantle melting dynamics, lithospheric thinning and rupture, and interaction with the crust as shown in previous studies^28–35^. Most recently a companion manuscript^36^ used the dataset to constrain the mineralogy and lithology of the mantle from which the magmas of the mid-Norwegian magmas formed. The pXRF data are particularly helpful for examining stratigraphic variations within the drilled lava successions. For instance, basalts recovered from depths shallower than 210 m below sea floor (mbsf) in Hole U1574A (Fig. 5; referred as unit Va in ref. ^16^) become progressively more primitive shallowing upwards that may record a recharge of the magmatic chamber at depth. Discrete pXRF data can also be used as calibration data for large high-resolution dataset acquired with core scanning facilities^46^.

## Technical Validation

The accuracy of the new data is validated through analyses of international reference standards and laboratory in-house standards together with the unknowns. The values obtained for these standards and a comparison with literature values (GeoREM, http://georem.mpch-mainz.gwdg.de/) can be found the new data file^19^.

The major element compositions measured for reference materials BCR-2, BHVO-1, BHVO-2, BIR-1, and RGM-1 are generally within 3% (relative deviation) of the expected GeoREM values, with exception of K_2_O values obtained at Aarhus University that deviates by up to 9%.

For the trace elements measured by ICP-MS, the analytical accuracies on W-2, BHVO-2, JB-2, and BCR-2 are better than 12% (relative deviation) for most elements in the three laboratories (GEUS, IACOS, Niigata) and often much better. The only exceptions are Li, Cr, Sn, Cs, Lu, Hf, Pb, U measured at Niigata University with relative deviations up to 20%.

Finally, the excellent match between ICP-MS and pXRF data on samples from the IODP Exp. 396 validates the calibrations of the pXRF measurements (Fig. 5).

## Data Availability

The data reported here are hosted in two separate files in the GEOROC repository at GFZ Data Services. One dataset provides new whole rock data on powder material (*n = *224) and pXRF data measured on the slip core surface (*n* = 381). This dataset can be downloaded as an excel file from this link: 10.5880/digis.2025.011. The second dataset provides a compilation of 563 entries of bulk rock major and/or trace element data, including published as well as the newly acquired whole rock powder data. This dataset is given as an *Expert Dataset* in the GEOROC repository and can be downloaded as an excel file via this link: 10.5880/digis.e.2025.005.

## Code availability

No custom code was used.
